# Factors Associated with COVID-19 Death in a High-Altitude Peruvian Setting during the First 14 Months of the Pandemic: A Retrospective Multicenter Cohort Study in Hospitalized Patients

**DOI:** 10.3390/tropicalmed8030133

**Published:** 2023-02-22

**Authors:** Fátima Concha-Velasco, Ana G. Moncada-Arias, María K. Antich, Carolina J. Delgado-Flores, Cesar Ramírez-Escobar, Marina Ochoa-Linares, Lucio Velásquez-Cuentas, Homero Dueñas de la Cruz, Steev Loyola

**Affiliations:** 1 Universidad Continental, Cusco 08000, Peru; 2 Dirección de Epidemiología e Investigación, Gerencia Regional de Salud (GERESA), Cusco 08200, Peru; 3 Facultad de Medicina, Universidad Peruana Cayetano Heredia, Lima 150135, Peru; 4 Instituto de Evaluación de Tecnologías en Salud e Investigación, EsSalud, Lima 15072, Peru; 5 Hospital Regional del Cusco, Cusco 08003, Peru; 6 Hospital Nacional Adolfo Guevara Velasco—EsSalud, Cusco 08002, Peru; 7 Hospital Antonio Lorena, Cusco 08001, Peru; 8 Doctorado en Medicina Tropical, Facultad de Medicina, Universidad de Cartagena, Cartagena de Indias 130014, Colombia

**Keywords:** COVID-19, risk factors, death, hospitalization, intensive care units, mechanical ventilator, high altitude, Cusco, Peru

## Abstract

Risk factors for COVID-19 death in high-altitude populations have been scarcely described. This study aimed to describe risk factors for COVID-19 death in three referral hospitals located at 3399 m in Cusco, Peru, during the first 14 months of the pandemic. A retrospective multicenter cohort study was conducted. A random sample of ~50% (1225/2674) of adult hospitalized patients who died between 1 March 2020 and 30 June 2021 was identified. Of those, 977 individuals met the definition of death by COVID-19. Demographic characteristics, intensive care unit (ICU) admission, invasive respiratory support (IRS), disease severity, comorbidities, and clinical manifestation at hospital admission were assessed as risk factors using Cox proportional-hazard models. In multivariable models adjusted by age, sex, and pandemic periods, critical disease (vs. moderate) was associated with a greater risk of death (aHR: 1.27; 95%CI: 1.14–1.142), whereas ICU admission (aHR: 0.39; 95%CI: 0.27–0.56), IRS (aHR: 0.37; 95%CI: 0.26–0.54), the ratio of oxygen saturation (ROX) index ≥ 5.3 (aHR: 0.87; 95%CI: 0.80–0.94), and the ratio of SatO_2_/FiO_2_ ≥ 122.6 (aHR: 0.96; 95%CI: 0.93–0.98) were associated with a lower risk of death. The risk factors described here may be useful in assisting decision making and resource allocation.

## 1. Introduction

Peru has the highest mortality worldwide [1]. Although Peru promptly implemented several non-pharmaceutical interventions at individual and population levels, such as physical distancing, use of masks, mandatory lockdowns, restrictions on gatherings, travel-related restrictions, and closure of non-essential services and workplaces, the SARS-CoV-2 spread rapidly throughout the country and the disease burden overwhelmed the health system [2]. The precarious organization of the Peruvian health system, healthcare disparities, shortage of medical oxygen, and the scarcity of Intensive Care Units (ICUs) and specialized healthcare personnel were factors that contributed to high mortality rates, particularly during the second wave of the COVID-19 pandemic [3,4,5].

A large proportion of deaths is expected in a hospital setting, since most of the attended patients are comorbid and have severe or critical diseases, thus requiring highly specialized care [6]. In this scenario, multiple biological and non-biological factors associated with death have been described; male, older age, comorbidities (such as hypertension and diabetes), hypoxemia, inflammation, availability, need for mechanical ventilation, and ICU admission [7,8,9].

Numerous investigations have evaluated factors associated with COVID-19 death in hospitalized patients; most of them have been conducted over a short period, and only a few of them have been conducted in Peru [10,11,12,13,14,15]. Of the studies carried out in Peru, one included information from three healthcare centers [12] and another described factors associated with ICU admission among hospitalized patients in a high-altitude setting [10]. Peru has been greatly affected by the pandemic. This situation resulted in an overwhelmed and fragmented health system that contributed to high and heterogeneous mortality rates between Peruvian cities and regions. The scarce characterization of death-related factors creates a knowledge gap and limits targeted efforts and decision making, as well as the implementation of population-specific interventions. The characterization of factors associated with COVID-19 death in a high-altitude Peruvian setting could contribute to the understanding of the COVID-19 mortality rate and address a knowledge gap. Here, we describe risk factors for COVID-19 death in three high-altitude health centers in Cusco, Peru, during the first 14 months of the pandemic.

## 2. Materials and Methods

### 2.1. Study Design and Sites

A retrospective multicenter cohort study was conducted in adult (≥18 years old) hospitalized patients in three tertiary referral hospitals in the region of Cusco, Peru. Hospital Regional del Cusco (HRDC), Hospital Nacional Adolfo Guevara Velasco—EsSalud (HNAGV), and Hospital Antonio Lorena (HAL) are high-altitude healthcare facilities located in the capital of the Cusco region (13°31′30″ S 71°58′20″ W), at an altitude of 3399 m in the Peruvian Andes. These hospitals care for approximately 1.2 million people who live in the region and people who are referred from nearby localities [16]. Given the level of complexity and geographical location, the three hospitals were designated for the care of all suspected and confirmed COVID-19 cases in Cusco. According to the Regional Health Administration (GERESA) of Cusco, a total of 77,843 COVID-19 cases and 1321 deaths related to COVID-19 were recorded in 2020, resulting in a mortality rate of 9.7 per 10,000 inhabitants and a case fatality rate (CFR) of 1.7%. By 2021, a total of 79,654 cases and 2991 deaths were recorded, resulting in a mortality rate of 22.0 per 10,000 inhabitants and a CFR of 3.8% [17].

The research and its secondary data analysis were approved by the Institutional Review Board of the Universidad Continental (019-2022-VI-UC) and by GERESA of Cusco. Hospitals also approved the study protocol and its procedures.

### 2.2. COVID-19 Death Definition

A COVID-19 death was defined as the death of a subject who met laboratory and/or imaging criteria. The laboratory criterion was composed of two components: (i) a positive reverse transcription-polymerase chain reaction (RT-PCR) and/or COVID-19 antigen test result using a nasopharyngeal swab, and/or (ii) the detection of IgM, IgM/IgG, or IgG by a serological COVID-19 rapid test using blood or serum/plasma. The subject had to meet at least one component of the laboratory criteria within 21 days before or after the hospital admission. This time frame was used because most life-threatening pulmonary complications occur in that period [18,19]. The imaging criterion was also composed of two components, and the subject had to meet at least one of them; (i) a score of at least 4 on the last computed tomography (CT) scan of the lungs according to the COVID-19 reporting and data system (CO-RADS) classification [20,21], and (ii) pulmonary infiltrate compatible with the “typical appearance” of COVID-19 pneumonia on the last CT scan [21].

### 2.3. Data Sources and Collection

Medical records of all deaths, including those caused by or related to COVID-19, are systematically and routinely recorded in the National Death Registry Information System (SINADEF; https://bit.ly/3hjRbOA; accessed on 1 July 2021) within the first 24 h, as required by Peruvian regulations. A retrospective search was conducted on 1 July 2021 in the SINADEF database listing U07.1 and/or U07.2 (International Classification of Diseases (ICD), 10 revision), and/or with any term related to COVID-19, including “COVID”, “SARS”, and/or “pneumonia”. Terms were included as part of the selection criteria since COVID-19 deaths may not have been correctly recorded using ICD-10 codes, but may have included contributing conditions that can later be used to correctly classify the record [22,23]. The search was restricted to hospitalized adult subjects with residence in Cusco who died between 1 March 2020 and 30 June 2021 in any of the three hospitals.

**Figure 1:**
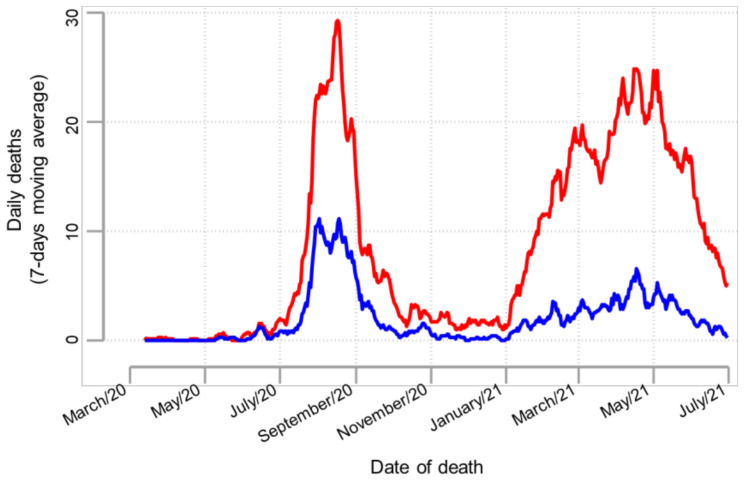
Epidemiological curves by date of death. The curve in red was constructed using the dates of death registered in 2674 records, and the curve in blue using the dates registered in 1225 records. These latter records were randomly selected from the 2674 records.

**Figure 2:**
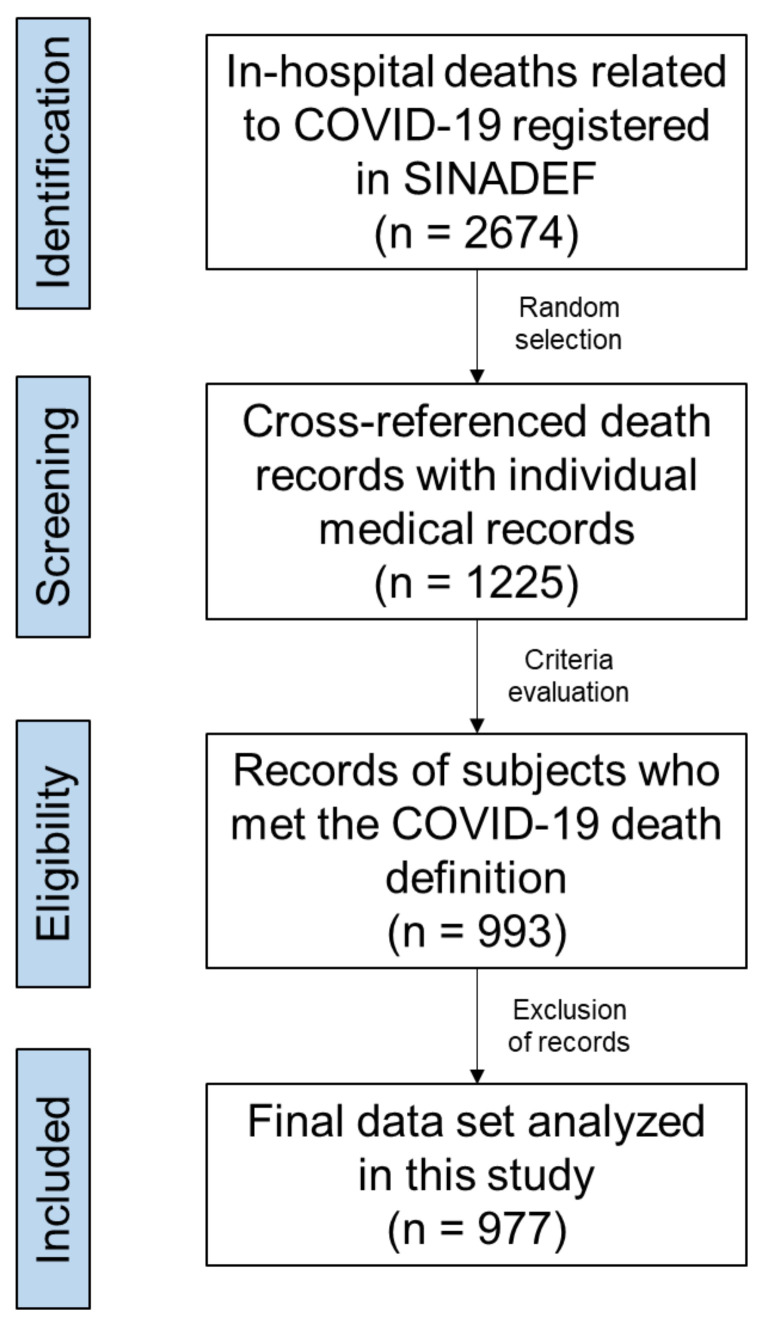
Flowchart of death record identification and selection process.

The search resulted in 2674 death records, of which 1225 (45.8%) were selected by a simple sampling method using random numbers generated in Epidat v4.1. To assess a potential selection bias associated with sampling, epidemiological curves (Figure 1) were constructed and compared using the dates of death registered in all records (*n* = 2674) and the selected records (*n* = 1225). As the pattern of deaths was comparable, it is reasonable to assume that there was a low risk of systematic bias introduced by sampling. Then, selected records were cross-referenced with individual medical records at each hospital by research-trained personnel for full data extraction (Figure 2). Using the definition of COVID-19 death described above, a total of 993 subjects who met the laboratory and/or imaging criteria were identified (Figure 2). However, sixteen records were excluded because subjects arrived dead at the hospital and therefore were not hospitalized (*n* = 15), or because records had incomplete data (*n* = 1). In Table 1, information on the 977 subjects is summarized according to the COVID-19 definition of death used in this study.

Epidemiological, demographic, and clinical data were extracted from medical records by research-trained physicians and nurses using a standardized form. All entries were de-identified to preserve patient confidentiality, and then the quality control was assessed by two research-trained physicians. In case of discrepancies, a third research-trained physician adjudicated the difference.

### 2.4. Outcome

The outcome of interest was time to death among hospitalized COVID-19 cases. The start and end of the follow-up time were defined by the patient’s admission to the hospital (time 0) and death, respectively.

### 2.5. Covariates

Demographic and clinical variables were collected at the hospital admission from patients’ medical records and then evaluated as predictors of death. Age (years), sex, and comorbidities, including cardiovascular disease (includes hypertension), diabetes, chronic lung disease, chronic neurological and neuromuscular disease, liver disease, cancer, and immunodeficiency (includes HIV/AIDS), were collected. COVID-19 disease severity (moderate, severe, or critically severe) was defined as described elsewhere [24], and registered symptoms such as respiratory distress, malaise, cough, fever (axillary temperature ≥ 37.3 °C) or chills, headache, chest pain, sore throat, muscle pain/aches, irritability or confusion, nasal congestion, arthralgia, nausea or vomiting, diarrhea, and abdominal pain were also collected. Data such as respiratory rate (breaths per minute), heart rate (beats per minute), the respiratory rate–oxygenation (ROX) index [25], the oxygen saturation to fraction of inspired oxygen ratio (SatO_2_/FiO_2_) [26], ICU admission, invasive mechanical ventilation, time elapsed from symptom onset to hospitalization, and time from hospitalization to death were also collected.

### 2.6. Statistical Analysis 

Categorical variables were described as *n* (%), and continuous variables as median and interquartile ranges (IQR). Three continuous variables were categorized as follows: age, <60 and ≥60 years; ROX index, <5.3, ≥5.3 to <13.5, and ≥13.5; and SatO_2_/FiO_2_, <122.6, ≥122.6 to <286.8, and ≥286.8 [27]. To account for potential changes in the epidemiology of COVID-19 deaths during the study period, we defined five time periods as follows: period I (1 March to 31 July 2020), period II (1 August to 31 October 2020), period III (1 November 2020, to 31 January 2021), period IV (1 February to 30 April 2021), and period V (1 May to 30 June 2021). These periods captured the temporal changes in the incidence of COVID-19 in the Cusco region [17]. Specifically, according to local health authorities, periods II and IV corresponded to the first and second waves of the COVID-19 pandemic, respectively [17].

The Fisher’s exact and Kruskal–Wallis with ties tests were used to assess differences between patient characteristics, comorbidities, symptoms, signs, ICU admission, invasive mechanical ventilation, and times from symptom onset to hospitalization and from hospitalization to death across defined periods. Cox proportional-hazard models were used to explore risk factors associated with COVID-19 death, and the Kaplan–Meier estimator was also used to assess differences in the outcome by ICU admission, invasive respiratory support, and disease severity. The multivariable-adjusted models included age and sex as confounders as described elsewhere [28], and in all models, the intra-group correlation of each hospital was specified. In addition, time-stratified and time-adjusted models were constructed to assess the robustness of the estimates. No other variables were included in the multivariable analysis to avoid overfitting. Crude and adjusted hazard ratios (HR) and their 95% confidence intervals (95%CI) were computed in Stata v17 (StataCorp. 2021. Stata Statistical Software: Release 17. College Station, TX, USA: StataCorp LLC). All tests were two-sided, and *p*-values < 0.05 were considered significant.

## 3. Results

A total of 977 death records from the same number of subjects who died from COVID-19 between May 2020 and June 2021 were analyzed. According to periods, 166 (16.9%) deaths were registered in period I, 329 (33.7%) in II, 68 (7.0%) in III, 299 (30.6%) in IV, and 115 (11.8%) in V.

### 3.1. Characteristics of the Study Population

The average age of the subjects who died from COVID-19 was 66.3 years (standard deviation: 12.9), and the proportion ≥60 years was 70.6% (Table 2). Throughout the study period, those aged ≥60 years, males, those not admitted to ICU, those not receiving invasive ventilatory support, and those hospitalized for severe illness were the largest groups (Table 2). The median time from symptom onset to hospitalization was 7 days (IQR: 5–10) and did not vary according to the periods (*p* = 0.298). The median time from symptom onset to death was 4 days (IQR: 2–9) and varied by periods (*p* < 0.001). Specifically, the median time for period I was 6 days (IQR: 3–11), 4 days (IQR: 2–9) for II, 5 days for III (IQR: 2–14), and IV (IQR: 2–9), and 3 days (IQR: 2–7) for V.

The most frequent comorbidities were cardiovascular disease (31.0%) and diabetes (20.7%), and the frequency of comorbidities was comparable across all periods (Table 3). Regarding symptoms, respiratory distress (89.5%), malaise (74.5%), cough (74.4%), and fever or chills (55.5%) were the most frequent. Interestingly, the clinical presentation varied significantly between periods (Table 3). However, four symptoms had no significant variation throughout periods: respiratory distress, cough, chest pain, and diarrhea (Table 3). The proportion of subjects with ROX index < 5.3 and SatO_2_/FiO_2_ < 122.6 was 35.6% and 34.1% (Table 3), respectively. The signs at hospital admission varied significantly between periods (Table 3).

### 3.2. Factors Associated with COVID-19 Death

In the bivariate analysis, a significantly greater risk of death was observed in those aged ≥60 years, and in periods II, IV, and V (vs. period I) (Table 4). In contrast, a significantly reduced risk of death was observed in males, in those admitted to the ICU, in those who received invasive respiratory support (vs. no), and in those with ROX index ≥ 5.3 (Table 4). 

**Figure 3:**
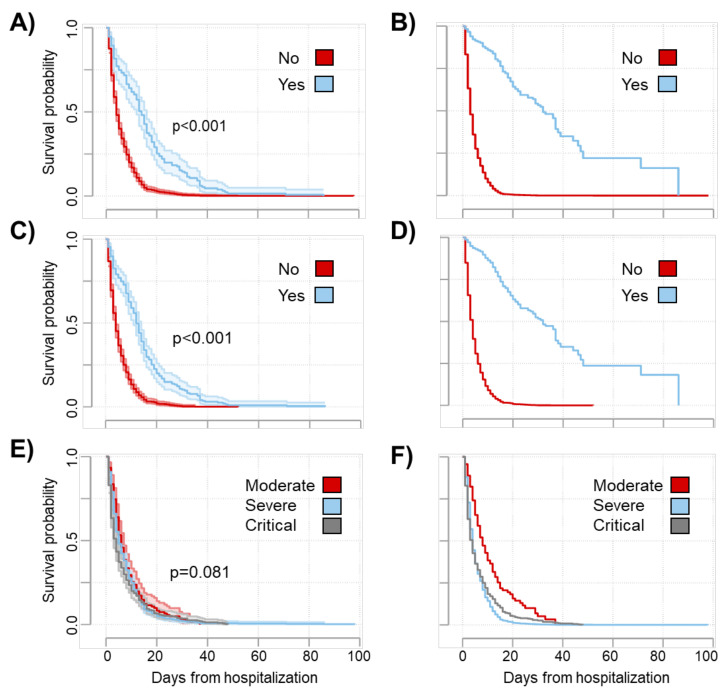
Kaplan–Meier curves and survival functions for deaths from COVID-19. Raw and adjusted survival functions were estimated by ICU admission (**A**,**B**), invasive respiratory support (**C**,**D**), and disease severity (**E**,**F**), respectively. Shaded areas represent the 95% confidence interval, and the log-rank test was used to test differences in estimated survival functions in raw analyses (**A**,**C**,**E**). Adjusted functions were constructed controlling age, sex, and periods.

The survival curves for hospitalized subjects by ICU admission, invasive respiratory support, and disease severity are shown in Figure 3. Specifically, those admitted to ICU and those who received invasive respiratory support exhibited a better probability of survival. The time-dependent probability of survival was comparable across all disease severity groups (Figure 3).

In multivariable models adjusted by age, sex, and periods, those with critical illness and those with cough or irritability/confusion had a significantly higher risk of death (Table 4), whereas those admitted to ICU, those who received invasive respiratory support, those with liver disease, those with ROX index ≥ 5.3 or SatO_2_/FiO_2_ ≥ 122.6, and those with chest pain, sore throat, or muscle pain/aches had a significantly lower risk of death (Table 4).

In the stratified multivariate models, having been admitted to the ICU and having received invasive ventilation was consistently associated with a significantly lower risk of death in nearly all time periods (Table 5). Regarding disease severity, there was an overall trend toward a higher risk of death for those with severe or critical diseases throughout all periods, except for period V. For this last period, the risk of death was lower, although it was not significant (Table 5). Furthermore, a ROX index ≥ 5.3 and SatO_2_/FiO_2_ ≥ 122.6 were both associated with a lower risk of death for almost all the evaluated periods.

## 4. Discussion

The evaluation of factors associated with death from COVID-19 is still of vital importance. Risk factor studies have found that age >60 years and oxygen saturation below 90% were associated with a higher risk of death [12,13,29,30,31]. In high-altitude settings, two Peruvian studies have suggested that saturation <80%, age between 40 and 60 years, comorbidities, and non-admission to ICU are factors associated with a greater risk of death [10,11]. Similarly, two Colombian studies conducted in a high complexity facility located at an altitude of 2640 m reported that older age and low SatO_2_/FiO_2_ ratio at admission were predictors of death [32,33]. Additionally, an Ecuadorian study suggested that in high-altitude settings, cases admitted to the ICU have better survival and disease progression [34]. In this multicenter cohort study conducted in three referral hospitals located in a high altitude setting and during the first 14 months of the pandemic, several variables such as demographic characteristics, ICU admission, invasive respiratory support, disease severity, comorbidities, and symptoms and signs at hospital admission were evaluated as risk factors in hospitalized patients. After controlling for sex, age, and periods, critical illness was associated with a higher risk of death, while ICU admission, invasive respiratory support, ROX index ≥ 5.3, and SatO_2_/FiO_2_ ratio ≥ 122 were associated with a lower risk of death. Overall, our results are similar to those that have been reported previously [10,11,32,33,34], and robust even in the stratified analyses performed in this study.

Early mechanical ventilation is associated with a reduced release of proinflammatory cytokines (such as IL-6, IL-8, and TNFα) that cause alveolar damage [35,36,37]. The availability of beds in critical care units plays a crucial role in patient care and early response [8,38]. In multiple regions of Peru, the hospital response capacity was substantially expanded and improved by increasing the number of ventilators and the number of available ICU beds; however, the increase was not sufficient, given the weak and fragmented nature of the healthcare system [2]. Our findings suggest that invasive respiratory support and ICU admission were both associated with a reduced risk of death from COVID-19 and improved survival probability. Despite not having formally assessed the response capacity of the hospitals studied across the time period, it is reasonable to hypothesize that greater availability of ventilators and ICU beds would have contributed to reducing deaths and improving the survival of hospitalized patients. As such, greater availability and better redistribution of resources could lead to a reduction in in-hospital mortality [8,38].

Physiological adaptations and genetic characteristics of individuals who live at high altitudes have been described as factors associated with lower mortality, higher probability of hospital discharge, and higher survival [34,39,40,41,42,43]. Notably, the most widely described factors that could account for the lower mortality rates in high-altitude populations are as follows: the hypobaric hypoxia in response to long-term exposure to high altitude and the resulting improved lung capacity, the reduced expression of the angiotensin-converting enzyme, and the higher levels of inflammatory cytokines (such as IL-6 y TNFα), hemoglobin, and erythropoietin [39,40,44,45]. It has also been reported that, compared to individuals residing in low-altitude areas, COVID-19 cases residing in high-altitude areas may present with low levels of fibrinogen and platelets, and disturbed electrolyte levels [46]. On the other hand, high-altitude environmental characteristics such as high ultraviolet radiation and low barometric pressure could play a role in virus survival and virus transmission, respectively [44,47]. However, it has also been suggested that infections, case-fatality rate, risk of death, and/or disease progression may not necessarily be associated with altitude, physiological adaptations, or both [48,49,50,51]. It is plausible to consider that the physiological adaptations, characteristics, and environmental exposures that have been outlined above influence the risks described here; however, their effect could not be estimated in the present study, given the absence of a comparison group. Future studies are needed to validate our findings through comparison with populations living in areas of varying altitudes.

The low oxygen saturation within the first 24 h of hospital admission predicts COVID-19 severity and death [52]. The utility of the oxygen saturation assessment could be affected by the oxygen–hemoglobin dissociation curve [53]; thus, the use of complementary parameters such as the ROX index and the SatO_2_/FiO_2_ ratio could be required [27]. The PaO_2_/FiO_2_ is the gold standard for the diagnosis of respiratory failure and ARDS [54]; however, this is an invasive method that is not always available in healthcare facilities. The ROX index and SatO_2_/FiO_2_ ratio are non-invasive clinical parameters that are easy to implement in emergency rooms. In this study, ROX ≥ 5.3 or SatO_2_/FiO_2_ ≥ 122 at hospital admission were associated with a lower risk of death. According to our findings, these cut-off values could be useful to discriminate cases with higher or lower risk of death. Future studies are needed to validate these findings.

The admission of patients with severe or critical illnesses demands highly specialized care. Based on our findings, patients with severe disease were the most frequently admitted to the hospital, followed by those with a critical illness. While the survival probability of those with severe or critical illnesses was comparable, the overall risk of death was only significantly higher in those with critical illnesses. This greater risk was only significant at the beginning of the first peak (period I) and the second peak (period IV) of the COVID-19 pandemic in Cusco. The inconsistency of this finding across all periods could be related to the lack of statistical power, in particular for periods III and IV, as well as to the low preparedness and collapse of the healthcare system in specific periods with a high number of COVID-19 cases. However, despite the significance, the risk of death was particularly greater for patients with critical illnesses.

The risk of death for hospitalized patients with or without diabetes or chronic lung disease was comparable. These findings differ from those reported elsewhere, where it is suggested that these conditions have an impact on mortality [15,55,56,57]. However, the lack of association observed here could be explained by the “diabetes/obesity paradox” and by the physiological readjustment during the critical stage of the disease [58]. Furthermore, we observed that patients with liver disease had a lower risk of death. However, this finding also differs from what has been previously reported [59,60]. It is important to note that several studies have shown contradictory results regarding the association of various comorbidities with COVID-19 death [15,29,61]. Overall, the lack of association between comorbidities evaluated here and COVID-19 death, as well as the observed association between liver disease and death, should be interpreted with caution given that the low number of individuals with comorbidities such as chronic lung or neurological disease, liver disease, cancer, and immunodeficiency could undermine the statistical power to detect differences, and the comorbidities may not have been correctly recorded or diagnosed. Hence, the associations between comorbidities and death described here could be biased. Further studies are needed to determine whether comorbidities, particularly in high-altitude settings, have a major impact on the risk of death from COVID-19.

This study has multiple limitations. First, this study is subject to limitations that are inherent to a retrospective study with secondary data analysis. Second, the analyzed data were collected at hospital admission; therefore, disease progression and post-admission events were not considered in the analyses. Third, the type of patient and their management could have been different at each healthcare institution. Although the multicenter design and the intra-group correlation control in all models can be considered strengths, it is still plausible to assume that results are affected by uncontrolled institutional characteristics and/or policies. Fourth, the hospital response capacity (such as the increase in ventilators and ICU beds) and the local dynamics of SARS-CoV-2 variants were not considered in the analysis. It is reasonable to expect that the risk of death would be lower in settings with higher availability of medical resources and that the risk would be greater in settings with the circulation of highly virulent variants that cause greater lung injury. The time-stratified analyses were performed under the assumption that unstudied or unknown conditions affect the estimates. Despite not having observed major differences in the estimates, further studies are needed to understand the effect of these conditions on the risk and survival of hospitalized patients. Finally, the diagnostic performance and the variable availability and supply of laboratory and imaging tests affect the definition of death used in this study. In Peru, RT-PCR was used as a diagnostic test during the first months of the pandemic. Later, RT-PCR was largely replaced by serology tests. RT-PCR and antigen tests have comparable diagnostic utility, whereas the diagnostic use of serology has been widely discussed in acute disease [62,63,64]. Furthermore, the use of CT scans in the diagnosis of COVID-19 is not perfect, and the identification of infiltrate could vary between observers [65,66,67]. Despite the use of a composite definition for COVID-19 death, the use of this definition did not overcome the bias inherent to each test used to construct the definition [68,69].

## 5. Conclusions

In summary, in this multicenter study in a high-altitude Peruvian setting during the first 14 months of the COVID-19 pandemic, we observed that ICU admission, invasive respiratory support, and ROX ≥ 5.3 or SatO_2_/FiO_2_ ≥ 122 at hospital admission were associated with a lower risk of death. In contrast, critical illness was associated with a higher risk of death. The risk factors described here may be useful in assisting clinical decision making and the allocation of specific resources for the care of COVID-19 patients admitted to the hospital.

## Figures and Tables

**Table 1:** Evaluation of the criteria used to define a COVID-19 death (*N* = 977).

	*n* (%)
Laboratory criteria	
RT-PCR and/or antigen test	389 (39.8)
Antibody test	345 (35.3)
RT-PCR and/or antigen test + antibody	12 (1.2)
Negative	231 (23.7)
Imaging criteria	
CO-RADS (≥4)	131 (13.4)
Pulmonary infiltrate	28 (2.9)
CO-RADS (≥4) + pulmonary infiltrate	485 (49.6)
Negative	333 (34.1)
Total positive components *	
One	393 (40.2)
Two	262 (26.8)
Three	318 (32.6)
Four	4 (0.4)

* The definition of COVID-19 death was constructed using two criteria (laboratory and imaging) composed of a total of four components. Description of each component is provided in Section “2.2 COVID-19 Death Definition”.

**Table 2:** Characteristics of subjects who died from COVID-19 in Cusco, 2020–2021.

	Total	Period I	Period II	Period III	Period IV	Period V	*p*-Value
	*N* (%)	*n* (%)	*n* (%)	*n* (%)	*n* (%)	*n* (%)	
Age (years)							
<60	287 (29.4)	53 (31.9)	80 (24.3)	12 (17.6)	106 (35.4)	36 (31.3)	0.006
≥60	690 (70.6)	113 (68.1)	249 (75.7)	56 (82.4)	193 (64.6)	79 (68.7)	
Sex							0.103
Female	301 (30.8)	38 (22.9)	99 (30.1)	21 (30.9)	104 (34.8)	39 (33.9)	
Male	676 (69.2)	128 (77.1)	230 (69.9)	47 (69.1)	195 (65.2)	76 (66.1)	
ICU admission							0.006
No	822 (85.9)	136 (82.4)	289 (90.3)	49 (74.2)	254 (86.4)	94 (84.7)	
Yes	134 (14.1)	29 (17.6)	31 (9.7)	17 (25.8)	40 (13.6)	17 (15.3)	
Invasive respiratory support							<0.001
No	654 (76.4)	123 (75.5)	271 (84.4)	31 (52.5)	164 (74.2)	65 (70.6)	
Yes	202 (23.6)	40 (24.5)	50 (15.6)	28 (47.5)	57 (25.8)	27 (29.4)	
Disease severity							0.193
Moderate	127 (13.0)	16 (9.6)	43 (13.1)	7 (10.3)	38 (12.7)	23 (20.0)	
Severe	498 (51.0)	89 (53.6)	158 (48.0)	35 (51.5)	165 (55.2)	51 (44.3)	
Critical	352 (36.0)	61 (36.7)	128 (38.9)	26 (38.2)	96 (32.1)	41 (35.7)	

**Table 3:** Clinical characteristics of subjects who died due to COVID-19 at hospital admission.

	Total	Period I	Period II	Period III	Period IV	Period V	*p*-Value
	*N* (%)	*n* (%)	*n* (%)	*n* (%)	*n* (%)	*n* (%)	
Comorbidities							
Cardiovascular disease	303 (31.0)	44 (26.5)	114 (34.8)	25 (37.3)	89 (29.8)	31 (27.0)	0.195
Diabetes	202 (20.7)	42 (25.3)	72 (22.0)	12 (17.9)	57 (19.1)	19 (16.5)	0.342
Chronic lung disease	50 (5.1)	5 (3.0)	26 (7.9)	3 (4.5)	12 (4.0)	4 (3.5)	0.081
Chronic neurological + neuromuscular disease	34 (3.5)	9 (5.4)	12 (3.7)	3 (4.5)	9 (3.0)	1 (0.9)	0.329
Liver disease	25 (2.6)	7 (4.2)	9 (2.8)	2 (3.0)	7 (2.3)	0 (0.00)	0.288
Cancer	24 (2.5)	2 (1.2)	15 (4.6)	1 (1.5)	5 (1.7)	1 (0.9)	0.050
Immunodeficiency	3 (0.3)	1 (0.6)	2 (0.6)	0 (0.00)	0 (0.00)	0 (0.00)	0.569
Symptoms							
Respiratory distress	855 (89.5)	142 (85.5)	304 (92.7)	57 (89.1)	253 (88.5)	99 (89.2)	0.154
Malaise	709 (74.5)	108 (65.1)	219 (66.8)	45 (69.2)	242 (86.1)	95 (84.8)	<0.001
Cough	727 (74.4)	119 (71.7)	238 (72.8)	49 (73.1)	227 (77.5)	94 (83.9)	0.104
Fever or chills	539 (55.5)	83 (50.0)	139 (42.6)	38 (56.7)	203 (68.1)	76 (66.1)	<0.001
Headache	429 (45.4)	59 (35.5)	104 (31.8)	26 (40.6)	172 (61.6)	68 (62.4)	<0.001
Chest pain	386 (39.7)	67 (40.4)	126 (38.5)	21 (31.3)	118 (39.7)	54 (47.0)	0.319
Sore throat	331 (34.1)	55 (33.1)	82 (25.1)	22 (32.8)	118 (39.7)	54 (47.4)	<0.001
Muscle pain/aches	237 (24.1)	20 (12.1)	43 (13.2)	19 (28.8)	109 (36.7)	46 (40.0)	<0.001
Irritability or confusion	151 (15.6)	22 (13.2)	38 (11.6)	13 (19.7)	59 (19.9)	19 (16.5)	0.046
Nasal congestion	144 (14.8)	9 (5.4)	36 (11.0)	10 (15.2)	58 (19.5)	31 (27.0)	<0.001
Arthralgia	123 (12.7)	6 (3.6)	13 (4.0)	12 (18.2)	65 (21.9)	27 (23.5)	<0.001
Nausea or vomiting	111 (11.3)	11 (6.6)	17 (5.2)	11 (16.7)	54 (18.2)	18 (15.7)	<0.001
Diarrhea	110 (11.3)	12 (7.2)	31 (9.5)	11 (16.7)	42 (14.1)	14 (12.2)	0.087
Abdominal pain	70 (7.2)	9 (5.4)	11 (3.4)	6 (9.1)	36 (12.1)	8 (7.0)	0.001
Signs							
ROX index							<0.001
<5.3	308 (35.6)	38 (24.2)	65 (22.8)	25 (45.4)	119 (45.1)	61 (59.2)	
≥5.3 to <13.5	329 (38.1)	75 (47.8)	128 (44.9)	15 (27.3)	84 (31.8)	27 (26.2)	
≥13.5	227 (26.3)	44 (28.0)	92 (32.3)	15 (27.3)	61 (23.1)	15 (14.6)	
SatO_2_/FiO_2_							<0.001
<122.6	314 (34.1)	39 (24.1)	67 (22.0)	27 (42.2)	125 (44.5)	56 (51.8)	
≥122.6 to <286.8	225 (24.5)	48 (29.6)	81 (26.6)	15 (23.4)	56 (19.9)	25 (23.2)	
≥286.8	381 (41.4)	75 (46.3)	157 (51.5)	22 (34.4)	100 (35.6)	27 (25.0)	

**Table 4:** Risk factors and hazard ratios of dying from COVID-19.

	HR (95%CI)	*p*-Value	aHR (95%CI)	*p*-Value
Age, years (Ref. < 60)				
≥60	1.31 (1.13–1.51)	<0.001	–	–
Sex (Ref. female)				
Male	0.82 (0.71–0.95)	0.007	–	–
ICU admission (Ref. no)				
Yes	0.38 (0.31–0.046)	<0.001	0.39 (0.27–0.56)	<0.001
Invasive respiratory support (Ref. no)				
Yes	0.37 (0.31–0.44)	<0.001	0.37 (0.26–0.54)	<0.001
Disease severity (Ref. moderate)				
Severe	1.09 (0.89–1.33)	0.393	1.09 (0.87–1.37)	0.471
Critical	1.22 (0.99–1.51)	0.059	1.27 (1.14–1.42)	<0.001
Periods (Ref. period I)				
Period II	1.27 (1.05–1.55)	0.015	–	–
Period III	0.89 (0.66–1.19)	0.420	–	–
Period IV	1.23 (1.01–1.49)	0.042	–	–
Period V	1.43 (1.12–1.82)	0.005	–	–
Comorbidities (Ref. no)				
Cardiovascular disease	0.99 (0.86–1.14)	0.889	0.96 (0.86–1.07)	0.476
Diabetes	0.99 (0.85–1.17)	0.986	0.99 (0.90–1.11)	0.972
Chronic lung disease	1.18 (0.87–1.59)	0.286	1.07 (0.94–1.22)	0.303
Chronic neurological + neuromuscular disease	0.90 (0.63–1.27)	0.533	0.87 (0.68–1.10)	0.242
Liver Disease	0.83 (0.53–1.29)	0.404	0.86 (0.75–0.99)	0.046
Cancer	1.36 (0.88–2.10)	0.160	1.41 (0.87–2.31)	0.166
Immunodeficiency	0.71 (0.23–2.21)	0.555	0.84 (0.49–1.02)	0.100
Symptoms (Ref. no)				
Respiratory distress	1.11 (0.89–1.34)	0.355	1.08 (0.87–1.35)	0.748
Malaise	0.93 (0.80–1.08)	0.354	0.88 (0.75–1.03)	0.144
Cough	1.14 (0.98–1.32)	0.101	1.09 (1.05–1.14)	<0.001
Fever or chills	0.95 (0.83–1.08)	0.425	0.93 (0.81–1.06)	0.331
Headache	0.97 (0.85–1.11)	0.706	0.94 (0.82–1.08)	0.070
Chest pain	0.89 (0.79–1.03)	0.113	0.87 (0.81–0.94)	<0.001
Sore throat	0.91 (0.79–1.04)	0.160	0.88 (0.82–0.96)	0.002
Muscle pain/aches	0.95 (0.81–1.10)	0.465	0.90 (0.82–0.99)	0.049
Irritability or confusion	1.21 (1.00–1.45)	0.045	1.16 (1.05–1.30)	0.005
Nasal congestion	1.12 (0.93–1.34)	0.235	1.03 (0.85–1.24)	0.464
Arthralgia	0.94 (0.78–1.15)	0.567	0.92 (0.75–1.13)	0.198
Nausea or vomiting	1.12 (0.91–1.37)	0.297	1.09 (0.85–1.39)	0.491
Diarrhea	0.90 (0.73–1.11)	0.319	0.88 (0.75–1.04)	0.143
Abdominal pain	0.98 (0.76–1.27)	0.864	1.04 (0.88–1.23)	0.642
ROX index (Ref. < 5.3)				
≥5.3 to <13.5	0.84 (0.72–0.99)	0.036	0.87 (0.80–0.94)	<0.001
≥13.5	0.83 (0.70–0.99)	0.042	0.82 (0.74–0.92)	0.001
SatO_2_/FiO_2_ (Ref. < 122.6)				
≥122.6 to <286.8	0.91 (0.76–1.09)	0.303	0.96 (0.93–0.98)	0.001
≥286.8	0.86 (0.74–1.01)	0.066	0.85 (0.80–0.91)	<0.001

HR: hazard ratio. aHR: adjusted hazard ratio by age, sex, and time periods.

**Table 5:** Risk factors and hazard ratios of dying from COVID-19 stratified by periods.

	Period I	Period II	Period III	Period IV	Period V
	aHR (IC95%)	aHR (IC95%)	aHR (IC95%)	aHR (IC95%)	aHR (IC95%)
ICU admission (Ref. no)					
Yes	0.42 (0.26–0.70) *	0.38 (0.34–0.43) *	0.47 (0.30–0.74) *	0.31 (0.26–0.38) *	0.44 (0.14–1.38)
Invasive respiratory support (Ref. no)					
Yes	0.43 (0.28–0.66) *	0.38 (0.33–0.40) *	0.39 (0.13–1.16)	0.30 (0.20–0.44) *	0.44 (0.22–0.89) *
Disease severity (Ref. moderate)					
Severe	1.09 (0.92–1.29)	1.09 (0.80–1.48)	1.21 (0.40–3.64)	1.25 (0.92–1.70)	0.94 (0.53–1.67)
Critical	1.55 (1.23–1.94) *	1.30 (0.99–1.68)	1.38 (0.50–3.77)	1.52 (1.32–1.74) *	0.86 (0.57–1.29)
ROX index (Ref. <5.3)					
≥5.3 to <13.5	0.97 (0.63–1.50)	0.76 (0.69–0.82) *	0.66 (0.40–1.09)	0.81 (0.75–0.88) *	1.27 (0.84–1.94)
≥13.5	1.09 (0.70–1.71)	0.71 (0.66–0.76) *	0.66 (0.53–0.83) *	0.74 (0.63–0.87) *	1.03 (0.86–1.23)
SatO_2_/FiO_2_ (Ref. <122.6)					
≥122.6 to <286.8	1.14 (0.90–1.44)	0.95 (0.87–1.03)	0.80 (0.39–1.62)	0.89 (0.79–1.01)	0.96 (0.81–1.14)
≥286.8	1.15 (1.07–1.23) *	0.74 (0.70–0.79) *	0.83 (0.52–1.32)	0.80 (0.74–0.87) *	0.99 (0.90–1.10)

aHR: adjusted hazard ratio by age and sex. * *p*-value < 0.05.

## Data Availability

The data presented in this study are available on request from the corresponding author.
